# Detecting Optic Disc on Asians by Multiscale Gaussian Filtering

**DOI:** 10.1155/2012/727154

**Published:** 2012-06-26

**Authors:** Bob Zhang, Jane You, Fakhri Karray

**Affiliations:** ^1^Department of Electrical and Computer Engineering, University of Waterloo, Waterloo, ON, Canada N2L 3G1; ^2^Biometric Research Center, and Department of Computing, Hong Kong Polytechnic University, Hong Kong

## Abstract

The optic disc (OD) is an important anatomical feature in retinal images, and its detection is vital for developing automated screening programs. Currently, there is no algorithm designed to automatically detect the OD in fundus images captured from Asians which are larger and have thicker vessels compared to Caucasians. In this paper, we propose such a method to complement current algorithms using two steps: OD vessel candidate detection and OD vessel candidate matching. The first step is achieved with multiscale Gaussian filtering, scale production, and double thresholding to initially extract the vessels' directional map of various thicknesses. The map is then thinned before another threshold is applied to remove pixels with low intensities. This result forms the OD vessel candidates. In the second step, a Vessels' Directional Matched Filter (VDMF) of various dimensions is applied to the candidates to be matched, and the pixel with the smallest difference designated the OD center. We tested the proposed method on a new database consisting of 402 images from a diabetic retinopathy (DR) screening programme consisting of Asians. The OD center was successfully detected with an accuracy of 99.25% (399/402).

## 1. Introduction

The optic disc is a vertical oval with average dimensions of 1.76 mm (horizontally) × 1.92 mm (vertically), and situated 3-4 mm to the nasal side of the fovea [1]. In fundus imaging the OD is usually brighter than its surrounding area and is the convergence of the retinal blood vessel network. Detection of the OD is useful in the diagnosis of glaucoma, optic neuropathies, optic neuritis, anterior ischemic optic neuropathy or papilledema, and optic disc drusen. It can also be used as a marker to help locate fovea/macula [2–4], as well as decide if the image is of the left or right eye. For diabetic retinopathy, detection of the OD assists physicians identifying neovascularization of the disc (NVD) in the advanced stage of DR, proliferative diabetic retinopathy (PDR). This makes the task of automatic OD detection both relevant and necessary. A distinction needs to be made between automatic OD detection and automatic OD boundary detection. The former refers to the location of the disc center, while the latter aims to segment the OD by detecting the boundary between the retina and the nerve head. Our work is detecting the OD center.

In the literature, OD detection can be categorized into various groups. The first group uses properties of the OD [5–8] such as high pixel intensity and its oval shape. Morphology [9] is also used where the OD center is the center of the brightest connected object found by thresholding an intensity image. References [10–12] applied template matching to locate the center. Osareh's et al. [10] template was the average gray-level of 25 normalized images, Lalonde et al. [11] used the Hausdorff-based template, and Youssif et al. [12] employed a Vessels' Directional Matched Filter. Two different kinds of transforms, Hough [13–15] and Watershed [16], have also been applied to locate the edges of the OD and subsequently its center. Supervised learning is another group consisting of feature extraction, and classification with a Bayesian [4] or k-NN [2, 3] classifier. A geometric model was built in [17] to represent the main retinal vessels which pass the OD center. Fuzzy convergence developed by Hoover and Goldbaum [18] determined the originating vessel map convergence point near the OD center. Even though the OD features and characteristics are well defined in fundus images, the task of automatically detecting its center is still challenging.

**Figure 1:**
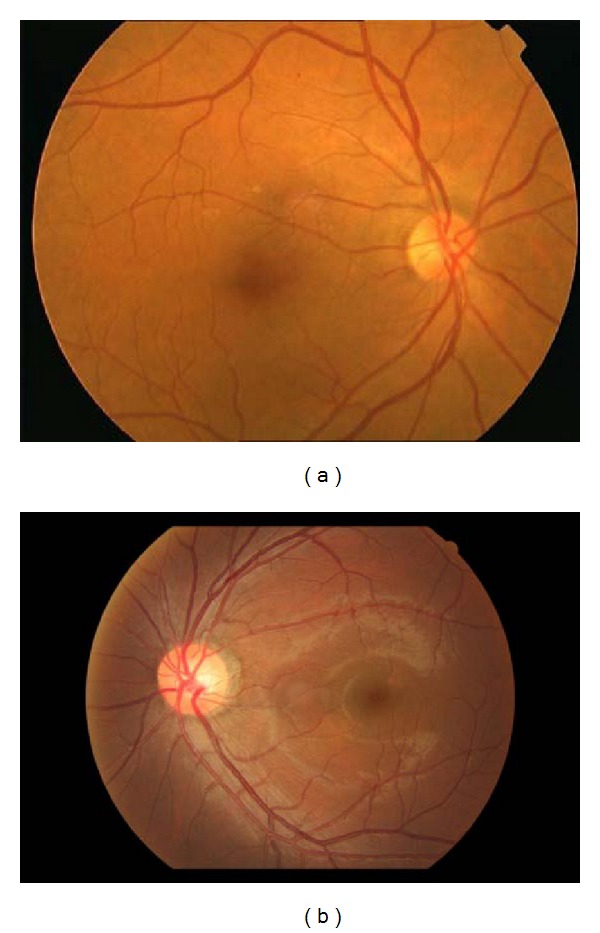
Comparison of Caucasian retina (a) to Asian (b).

**Figure 2:**
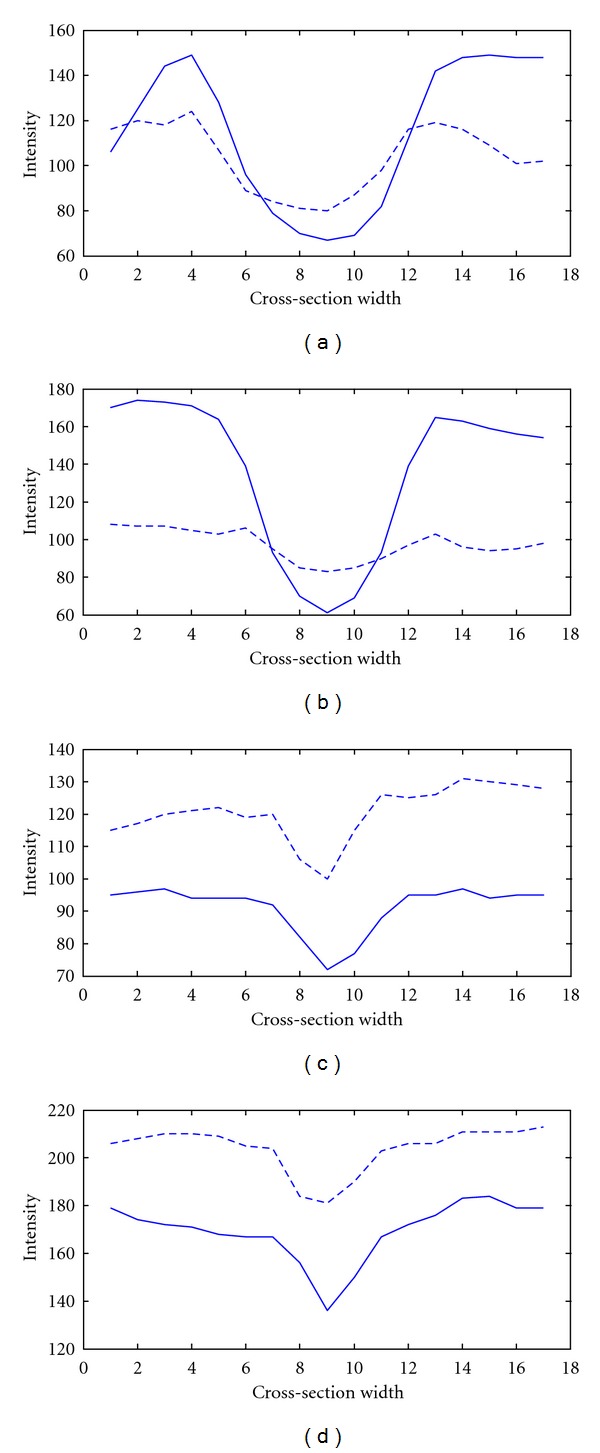
Thick (a) and (b); thin (c) and (d) vessel cross-sections. The solid line is Asian while the dotted comes from Caucasian.

Ethnicity affects the OD shape. A study conducted by [19] measuring disc area, cup volume, maximal cup depth, and vertical cup-disc ratio showed that Asians have higher values in these properties compared to Caucasians. This can be clearly seen in Figure 1 where (a) [20] is Caucasian while (b) is Asian, taken from our database. Further studies [21–24] demonstrated that with an increase in size of the OD, blood vessels (both arteries and veins) increase in thickness, where venular widening is linked with progression of DR [24] and narrowing of the arteriolar is associated with the risk of diabetes [24]. The increase in vessel thickness caused by an enlarged OD can be seen in Figure 2, which shows the cross-sections of both thick ((a)-(b)) and thin ((c)-(d)) vessels, taken from two individuals. The dotted line is Caucasian, while the sold line is Asian. Generally speaking for thick vessels the cross-section of Asians is about 3-4 pixels wider than Caucasians. On the other hand, for thin vessels the difference is around 1-2 pixels. In order to achieve a fair assessment we examined vessels only in the OD, comparing with the same orientation and grade of DR.

Most of the current algorithms working on OD detection are from Western countries; therefore, we can safely assume that the fundus images collected in their database are of Caucasians. Hence, there is a need to accurately detect the OD from Asians as state-of-the-art algorithms do not take into account the increased OD size and its subsequent consequences. This paper proposes such a method of dealing with Asian OD using both vessel and intensity information [25] to complement existing algorithms. In the first step, a vessels' directional map representing the OD vessel candidates is calculated using multiscale Gaussian filtering with scale production and double thresholding. This accounts for the thicker vessels of Asians. A VDMF template (with bilinear interpolation) is matched to each OD vessel candidate as part of the second step [12]. The pixel candidate having the least difference with the template is assigned the OD center.

The remainder of this paper is organized as follows.  Section 2 describes the material used, Section 3 presents the proposed method, and experimental results are relayed in Section 4. These results are discussed in Section 5 and a conclusion is given in Section 6.

## 2. Material

**Figure 3:**
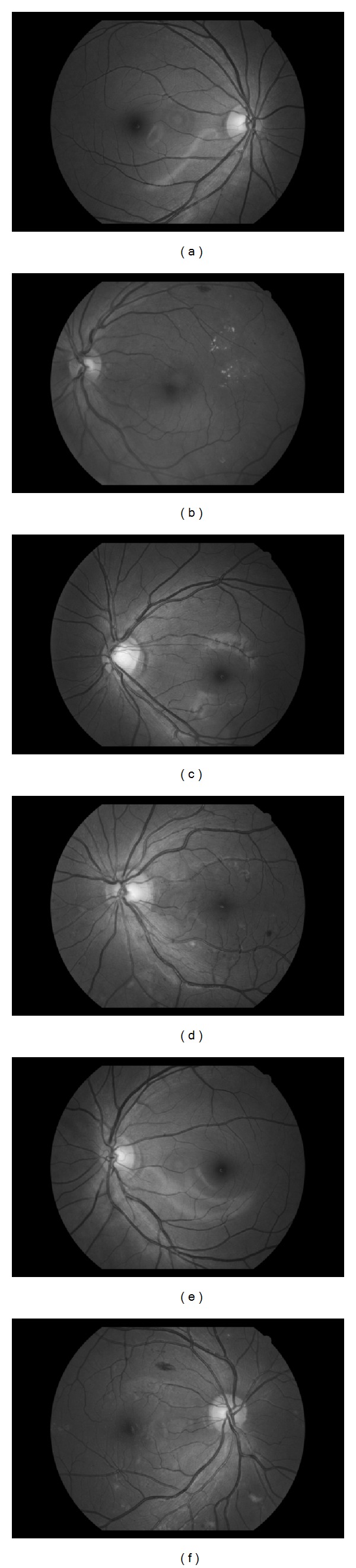
(a), (c), and (e) Normal fundus image from HIT. (b), (d), and (f) Fundus diagnosed with moderate NPDR from HIT.

We constructed a new database obtained from a DR screening programme in Harbin, China. The patients' consent was obtained according to the Declaration of Helsinki and that the Ethical Committee of the Institution in which the work was performed has approved it. This database will be referred to as the HIT database. HIT consists of 402 images, broken down into 46 normal and 356 pathological (all with DR). The 356 DR images were further divided into 181 mild nonproliferative diabetic retinopathy (NPDR), and 175 moderate NPDR, classified based on [26]. The images were captured in digital form using a Canon CR-DGi Non-Mydriatic Retinal Camera at 45° field of view (FOV). The size of each image is 1936 × 1288 pixels with 24 bits and in compressed JPEG format.  Figure 3 shows examples of images from HIT; the ones on the left are healthy retinas while the others have moderate NPDR. The OD center in each image of the database was manually segmented by the first author.

## 3. Method

**Figure 4:**
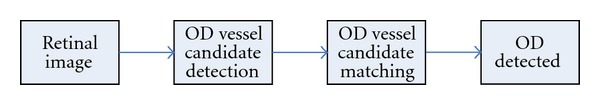
Steps of the proposed method shown in rectangles and its control flow given by the arrow.

As mentioned above, the proposed method based on Youssif et al. [12] consists of two steps (illustrated in Figure 4), OD vessel candidate detection and matching. The following section explains each step in more detail.

### 3.1. OD Vessel Candidate Detection

Multiscale Gaussian filtering is based on matched filters first proposed in [27] to detect vessels. It makes use of the prior knowledge that the cross-section of vessels can be approximated by a Gaussian function. Therefore, a Gaussian-shaped filter can be used to “match” the vessels. The idea of multiscale allows more than one scale to be used which can match vessels of various widths.   The multiscale Gaussian filter is defined as

(1) fi(x,y)=12πsiexp⁡⁡(−x22si2)−m, |x|≤t·si,  |y|≤Li2,

where *s*_*i*_ represents the scale of the filter; $m=\left(\int_{-ts}^{ts}\left(1/\sqrt{2\pi }s_{i}\right)\exp\left(-x^{2}/{2s_{i}}^{2}\right)dx\right)/\left(2ts_{i}\right)$ is used to normalize the mean value of the filter to 0 so that the smooth background can be removed after filtering; *L*_*i*_ is the length of the neighborhood along the *y*-axis to smooth noise; *t* is a constant and is usually set as 3 because more than 99% of the area under the Gaussian curve lies within the range of [−3*s*_*i*_, 3*s*_*i*_]. The parameter *L*_*i*_ is also chosen based on *s*_*i*_. When *s*_*i*_ is small, *L*_*i*_ is set relatively small, and vice versa. In the actual implementation *f*_*i*_(*x*, *y*) will be rotated to detect the vessels of different orientations.

The response of multiscale Gaussian filtering can be expressed by

(2) Ri(x,y)=fi(x,y)∗im(x,y),

where im(*x*, *y*) is a normalized green channel image and ∗ denotes convolution. The scale production is defined as the product of filter responses at two scales *i* and *j*:

(3) P(x,y)=Ri(x,y)·Rj(x,y).

**Figure 5:**
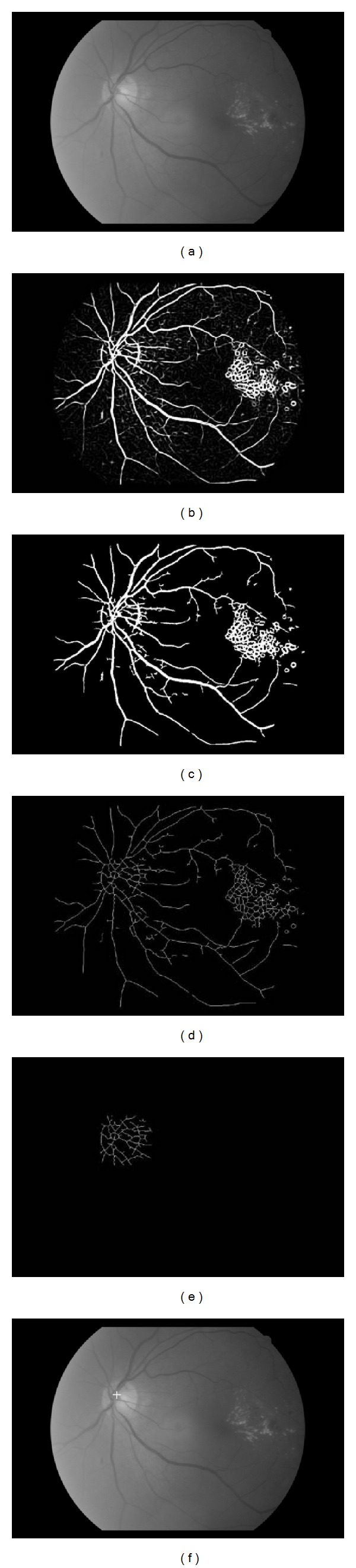
Steps of the proposed method applied to a fundus image (a). (b) is the scale production of (a). The result of double thresholding on (b) is (c). The thinned vessels of (c) is (d). (e) is the OD vessel candidates after removing pixels with low intensities. The detected OD center is illustrated in (f) by a cross.

Double thresholding is then applied to *P*(*x*, *y*) to generate a binary image where a one-pixel-wide center line of the vessel is detected using morphological thinning. The vessels' directional map is calculated by finding the corresponding orientation that produced the maximum response with *f*_*i*_(*x*, *y*) (use of the vessel feature). This map is thinned by multiplying with the center-line vessel. Using the notation that the OD has higher pixel intensities (use of the intensity feature) than its surrounding retinal background, any pixels less than 0.9 in im(*x*, *y*) are removed. A 51 × 51 neighborhood of each remaining pixel is extracted in order to better represent the OD vessels. This results in the OD vessel candidates. Figures 5(a)–5(e) illustrates these steps using an example. In some situations hard exudates may also be part of the OD vessel candidates since their pixel intensity is also high. However, these objects are not made of vessels and will be removed in the following step. In [12] normalization of luminosity and contrast were applied to the retinal images, with its vessels extracted using a one scale 2D Gaussian Matched Filter [27].

### 3.2. OD Vessel Candidate Matching

**Figure 6:**
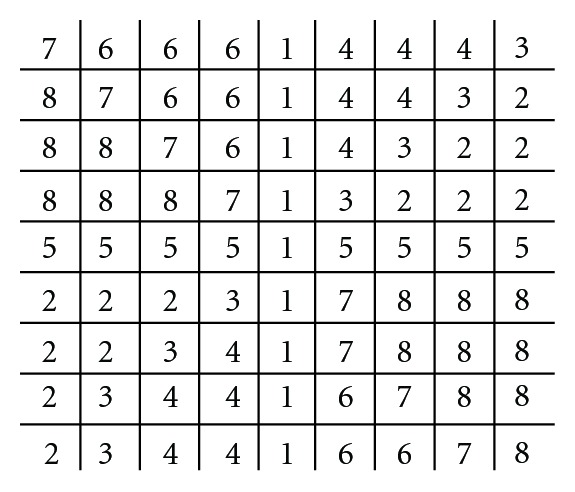
A vessels' directional matched filter designed for HIT.

We define a 9 × 9 template as the VDMF shown in Figure 6. Each value in this template represents a different orientation (rad), 1  (*π*/1), 2  (*π*/2),…, 8  (*π*/8), where 8 are used instead of 12 [12]. In order to account for the various sizes of vessel maps in the OD, bilinear interpolation was employed to restructure the template into 61 × 21 and 121 × 41. The values and sizes are specifically tuned for HIT. Reference [12] employed 4 different template dimensions suited for their database. Each of the two templates is matched to the candidates with an absolute difference calculated. The candidate pixel with least accumulated difference is assigned the OD center.  Figure 5(f) shows the final result.

## 4. Experimental Results

The key parameters in our experiments are set as follows: *s*_1_ = 1.5, *s*_2_ = 1.8, *s*_3_ = 2.0, and *s*_4_ = 2.4, with corresponding *L*_1_ = 9, *L*_2_ = 9, *L*_3_ = 13, and *L*_4_ = 13, and 8 orientations (refer to (1)). The scale production (see (3)) of *s*_1_ and *s*_2_ is combined along with the result of *s*_3_ and *s*_4_ using logical OR after double thresholding. These parameters were chosen based on our experimental experience. It took 29 secs to process each image using a 2.40 GHz Intel Centrino Pro with 2 GB RAM. In order to improve the computation time of the proposed method every image in HIT was resized by 0.5 to 968 × 644 pixels. In the literature, the detected OD center is considered correct if it is positioned within 60 pixels of the manually identified center [12, 17, 18]. However, the images [12, 17, 18] used were 605 × 700 pixels.

In order to compensate for the larger images in HIT, the distance from the manually identified center to the detected OD center is increased to 80 pixels. For completeness we have included results using both 80 and 60 pixels and compared the proposed method to Youssif et al. [12] as well as single scale. The original parameters stated in Youssif et al. [12] were used. Single scale [27], as the name suggests, uses one *s*_*i*_ in (1) instead of many (as is the case with multi-scale). This is combined with VDMF to detect the OD and evaluated against the proposed method. These can be found in Tables 1 and 2, respectively. Columns 2–5 in Tables 1 and 2 represent the number of normal, mild NPDR, moderate NPDR, and the total number of images. With 80 pixels the OD center in all of normal was detected. Only 1 and 2 were missed in mild and moderate NPDR which lead to an overall success rate of 99.25% (399/402). As for Youssif et al. [12], 2 were not detected in normal, 4 in mild NPDR, and 4 again in moderate NPDR. The result of single scale can be found in the last row, where 44, 178, and 170 were detected, respectively, for each of the three DR classes. Using 60 pixels (see Table 2), 1 was missed in all of normal, 4 from mild NPDR, and 4 in moderate NPDR. The success rate was 97.76% (393/402). Obviously, by raising the standard from 80 to 60 pixels the success rate is bound to drop which is the situation here. In cases where the OD center failed to be detected, one of the main reasons was low contrast exhibited by the image. We admit in such cases our method may fail. Youssif et al. [12] on the other hand failed to detect 9, 13, and 16 from the three classes, while single scale missed 8 from normal, 12 from mild NPDR, and 18 in moderate NPDR.

**Figure 7:**
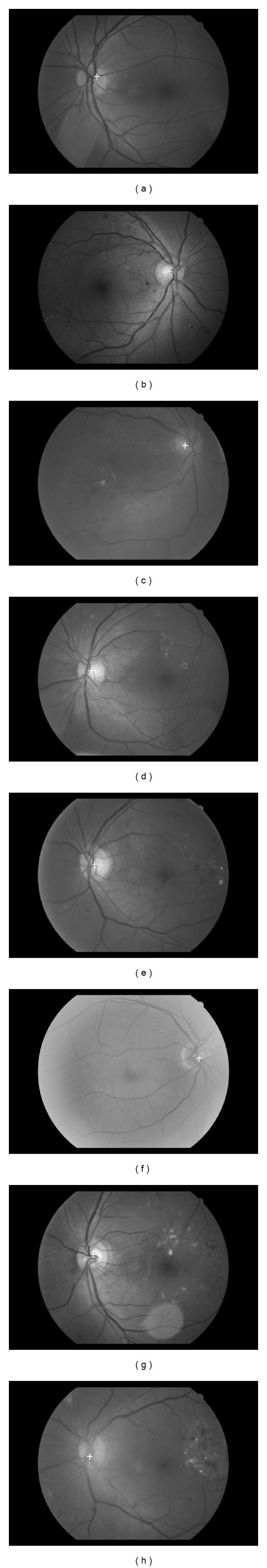
Results of the proposed method where a white cross represents the detected OD center.

Table 3, which has the same format as the previous two, illustrates the average distance between the estimated and manually identified OD centers. In this table the average distance of normal is 27.9 pixels, 28.8 for mild NPDR, 31.1 in the case of moderate NPDR, and 29.3 for the average of all three. This compares to averages of 35.7 using Youssif et al. [12] and 40.1 with single scale.  Figure 7 shows the OD detection results of the proposed method on images from HIT.

## 5. Discussion

**Figure 8:**
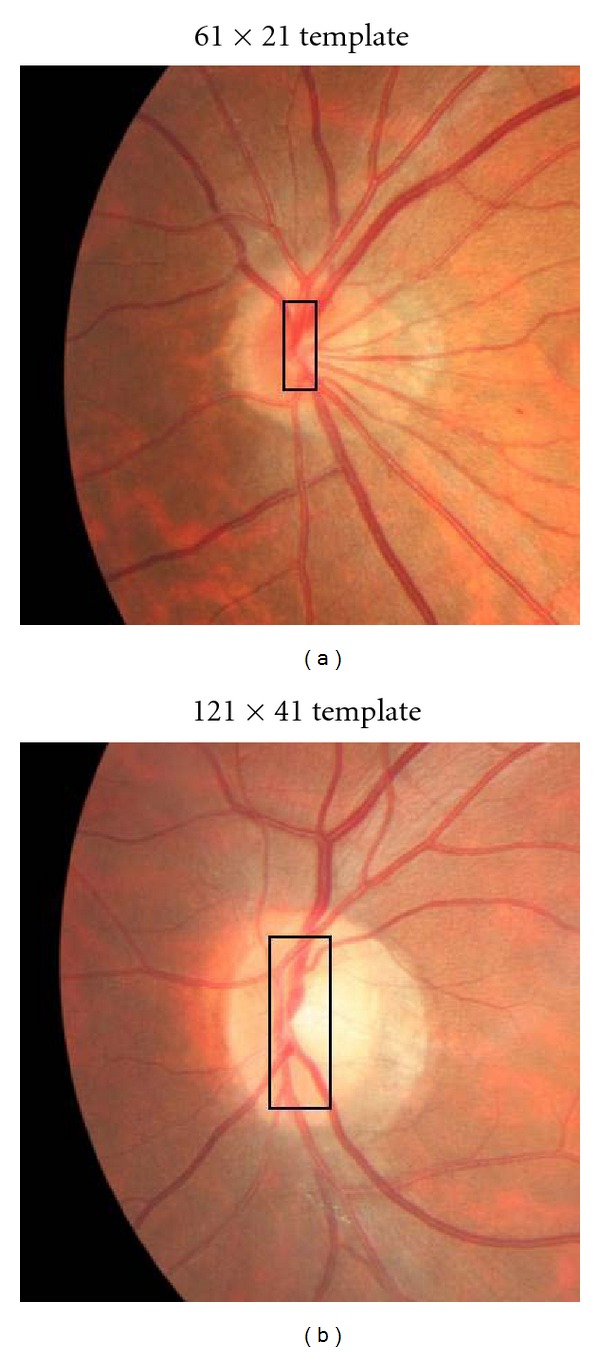
A small vessel map is covered with the 61 × 21 template (a). Medium-to-large maps are covered using the 121 × 41 template (b).

The variables in (1) were chosen based on extensive experiments. Because there is no ground truth for the vessel maps, our criteria is judged on the visual result (binary image of segmented vessel map) of several images. We tested *s*_*i*_ = 0.5 ~ 3 and found the current parameters in Section 4 to give the best result. As for the two template sizes in Section  3.2, 61 × 21 corresponds to covering a smaller vessel map in the OD, while 121 × 41 is for maps of medium-to-large dimensions. These are shown in Figure 8.

**Figure 9:**
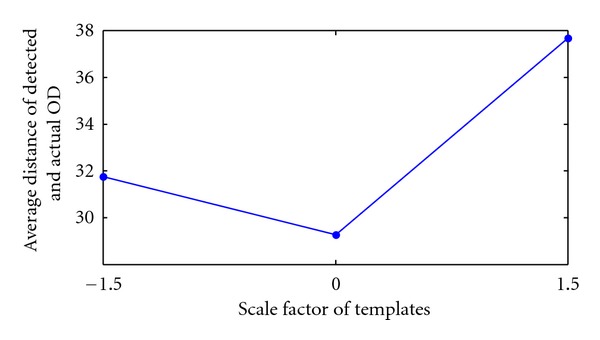
The result of average distance compared with various scale factors applied to the templates. From right to left, the first point is +1.5, next is the current template size, and the left point is −1.5.

Figure 9 depicts the result of increasing/decreasing the template size by a factor of ±1.5. In total there were 4 templates and 3 pairings, 61 × 21 with 121 × 41 (original), 61 × 21 with 151 × 51(+1.5), and 61 × 21 with 101 × 33(−1.5). Each pair corresponds to a point in Figure 9 which plots the average distance of detected and actual OD. The left (31.8 pixels) and right (37.7 pixels) points in Figure 9 are based on a scale factor of −1.5 and +1.5, respectively. This scale factor was chosen specifically for the HIT database. As can be seen from Figure 8 anything greater or smaller would not cover the OD efficiently affecting OD vessel candidate matching. The smallest distance was achieved with the current templates discussed above (see Section 3.2) and shown in Figure 9 as the central point.

The results from Table 1 show that even if the proposed method, Youssif et al. [12] and single scale achieve similar accuracy using 80 pixels (7 image difference), their gap is much wider with 60 pixels (29 image variation). Subsequently, the average distance between actual and estimated OD centers of the proposed method and single scale is close to 11 pixels, while for Youssif et al. [12] it is slightly less than 6 pixels. This shows the proposed is more accurate at OD detection compared to the others. Note in Tables 1 and 2 Youssif et al. [12] and single scale detected the same number of OD. We believe this to be the case because both methods incorporate single-scale vessel extraction in order to locate OD vessel candidates. 

**Figure 10:**
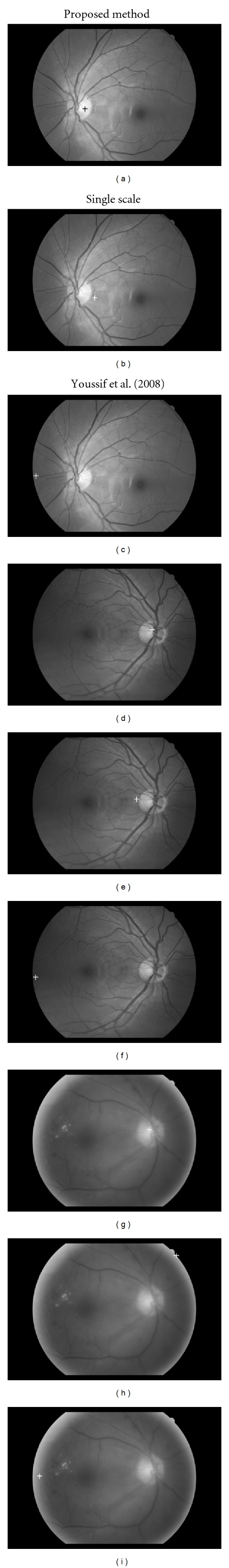
Visual comparison of the OD detection results (proposed method with others).

Figure 10 illustrates a sample of the OD detection results of the proposed method, single scale, and Youssif et al. [12]. In total three images are used, one from each DR group. The columns in Figure 10 represent the different OD detection methods. In this figure you can clearly see that the proposed method was able to detect the OD (all marked with a white cross except (a) which used a black cross), while the other two methods failed. Each of the retinal images in this figure is composed of a vascular map that consists of both thick and thin vessels. Hence, multiscale with VDMF was able to triumph, while single scale and Youssif et al. [12] both failed, due to its inability to match both thick and thin vessels found in Asians as well as account for a larger OD. This underlines the necessity of the proposed method at OD detection on Asians.

Other advantages of the proposed method include its capacity to calculate a vessels' directional map (in OD Vessel Candidate Detection) implicitly, while extracting the vessels without any additional algorithms as needed in [28, 29]. Also, our proposed method does not make any assumptions about the location of the OD in the image. In computer-aided diagnosis of HIT, vessel extraction is a first step. Hence, the time needed to extract the vessels can be discounted.

## 6. Conclusion

This paper presented a method for automatic detection of Asian OD to complement existing algorithms using two steps, OD vessel candidate detection, and OD vessel candidate matching. The study was deemed necessary since an Asian OD is typically larger than Caucasians and has thicker vessels. The proposed method makes use of the OD intensity information by removing low pixel values, and incorporates the vessel information in the form of a vessel's directional map. The use of multiscale Gaussian filtering at extracting both thin/thick vessels and the VDMF can be attributed to the high OD detection results seen in Tables 1–3. These tables along with Figure 10 show that algorithms developed in the West cannot adequately deal with the added properties of an Asian OD. Also, the use of single scale and Youssif et al. [12] are insufficient to match the various widths of the vessel.

We have carefully examined the cases in which the proposed method failed to correctly detect the OD center. In the majority of failed detections, poor contrast, imaging artifacts, and the presence of pathology, or a combination of these factors have attributed to the problem. When contrast over the OD is low, this affects the result of OD vessel candidate detection. As part of the future work, the aspect of contrast enhancement will be integrated to deal with such cases.

## Figures and Tables

**Table 1:** OD detection result on HIT using 80 pixels as standard.

Number of images in each group				Total
	46	181	175	402

Method	Number correctly detected			

Proposed method	46	180	173	399
Youssif et al. [12]	44	177	171	392
Single scale	44	178	170	392

**Table 2:** OD detection result on HIT using 60 pixels as standard.

Number of images in each group				Total
	46	181	175	402

Method	Number correctly detected			

Proposed method	45	177	171	393
Youssif et al. [12]	37	168	159	364
Single-scale	38	169	157	364

**Table 3:** Average distance (in pixels) of detected and actual OD.

Number of images in each group				Total
	46	181	175	402

Method	Average distance between estimated and actual OD center (pixels)			

Proposed method	27.9	28.8	31.1	29.3
Youssif et al. [12]	33.5	36.1	37.6	35.7
Single-scale	40.0	36.1	44.3	40.1
